# Designing a Mobile e-Coaching App for Immigrant Informal Caregivers: Qualitative Study Using the Persuasive System Design Model

**DOI:** 10.2196/50038

**Published:** 2023-11-09

**Authors:** Shweta Premanandan, Awais Ahmad, Åsa Cajander, Pär Ågerfalk, Michal Dolezel, Lisette van Gemert-Pijnen

**Affiliations:** 1 Department of Informatics and Media Uppsala University Uppsala Sweden; 2 Division of Visual Information and Interaction Department of Information Technology Uppsala University Uppsala Sweden; 3 Department of Information Technologies Faculty of Informatics and Statistics Prague University of Economics and Business Prague Czech Republic; 4 Department of Psychology, Health, and Technology Faculty of Behavioral, Management and Social Sciences University of Twente Enschede Netherlands

**Keywords:** e-coaching, mobile health, mHealth, immigrant informal caregivers, designing app, persuasive system design, user needs, caregiver, app, design, users, aging, development, diversity, language barrier, inclusion, training, mental health, mobile phone

## Abstract

**Background:**

Informal caregivers are vital in caring for their family and friends at home who may have illnesses or disabilities. In particular, the demands for caregiving can be even more challenging for those with limited resources, support systems, and language barriers, such as immigrant informal caregivers. They face complex challenges in providing care for their relatives. These challenges can be related to sociocultural diversity, language barriers, and health care system navigation. Acknowledging the global context of the increasing number of immigrants is essential in designing inclusive mobile health apps.

**Objective:**

This study aims to investigate the needs of immigrant informal caregivers in Sweden and discuss the application of the Persuasive System Design Model (PSDM) to develop an e-coaching prototype. By addressing the unique challenges faced by immigrant informal caregivers, this study will contribute to the development of more effective and inclusive mobile health apps.

**Methods:**

The participants were considered immigrants and included in the study if they and their parents were born outside of Sweden. Through various channels, such as the National Association of Relatives, rehabilitation departments at municipalities, and immigrant groups, we recruited 13 immigrant informal caregivers. These immigrant informal caregivers were primarily women aged 18 to 40 years. Most participants belonged to the Middle Eastern region whereas some were from North Africa. However, all of them spoke Arabic. We used semistructured interviews to gather data from the participants in Arabic, which were translated into English. Data were analyzed using thematic analysis and discussed in relation to the extended PSDM. The needs of the caregivers were compared with the description of persuasive design principles, and a design principle was chosen based on the match. The PSDM was extended if the need description did not match any principles. Several brainstorming and prototyping sessions were conducted to design the mobile e-coaching app.

**Results:**

Immigrant informal caregivers have various needs in their caregiving role. They reported a need for training on the illness and future caregiving needs, assistance with understanding the Swedish language and culture, and help with accessing internet-based information and services. They also required recognition and appreciation for their efforts, additional informal support, and easy access to health care services, which can be important for their mental health. The PSDM was adapted to the informal caregiving context by adding “facilitating conditions” and “verbal encouragement” as additional persuasive design principles. This study also presents the subsequent mobile e-coaching app for immigrant informal caregivers in Sweden.

**Conclusions:**

This study revealed important immigrant informal caregivers’ needs based on which design suggestions for a mobile e-coaching app were presented. We also proposed an adapted PSDM, for the informal caregiving context. The adapted PSDM can be further used to design digital interventions for caregiving.

## Introduction

### Background

Informal caregivers provide care for their relatives and friends in the long term, often without proper training or experience [1]. Consequently, informal caregiving can take a toll on the well-being of informal caregivers (hereafter referred to as caregivers) and impact their ability to continue providing quality care [2]. Approximately 80% of long-term care in Europe is believed to be provided by informal caregivers, with women accounting for approximately two-thirds of this care provision [3]. In Sweden, 1 in 5 individuals is an informal caregiver [4], which is expected to increase owing to Europe’s aging population. Hence, informal caregiving is crucial for traditional health care. It encompasses a broad range of support, including practical assistance such as domestic work and personal care, emotional support, and administrative tasks such as coordinating care and interacting with public authorities [5]. The impact of caregiving extends beyond the patients themselves, affecting the physical and psychological well-being of caregivers [6]. Many caregivers face a range of psychological challenges, including depression, anxiety, and posttraumatic stress [7]. Caregiving frequently brings about physical, emotional, and financial stress, impacting the caregiver’s well-being and ability to provide quality care [8]. Caregiving demands can be especially challenging for those with limited resources or support systems, such as immigrant informal caregivers.

Several studies have highlighted the prevalence of informal caregiving in Sweden and its impact on caregivers’ well-being, emphasizing the need for better support systems. Families are still the major providers of care for older adults in Sweden, suggesting that it is common to be an informal caregiver [9]. The study by Jegermalm [10] indicated that relatively few caregivers in Sweden have any kind of support aimed directly at them as caregivers. This lack of support is a critical issue that must be addressed to improve the well-being of informal caregivers. The research by Berglund et al [11] further supports this point, emphasizing that caregivers have worse perceptions of self-rated health and psychological well-being than noncaregivers in Sweden. Hence, it is important to recognize and support informal caregivers in Sweden. Caregivers in Sweden need recognition and support from the health care system and are interested in being considered as care partners [12]. Moreover, there are significant gender-related differences in the number of male and female caregivers based on different caregiving tasks [10]. In particular, studies have highlighted that women are more likely to be engaged in intensive caregiving, including personal care and other caregiving tasks. Simultaneously, males were more likely to provide practical assistance to mothers, neighbors, and friends. These gender-related differences highlight the pressing need to address caregivers’ support needs, particularly women, who are often engaged in intensive caregiving tasks. Overall, these findings suggest that better support systems for informal caregivers in Sweden are essential to ensure that the social care system is better equipped to meet the needs of both caregivers and care recipients.

There is a growing immigrant population in Europe. In Sweden alone, with a total population of approximately 10 million, 102,000 people immigrated in 2022 [13]. Owing to a tougher sociopolitical climate, immigrant caregivers were forced to care for themselves to a greater extent with limited support from authorities and other public support systems. Immigrant informal caregivers often face complex challenges in providing care for their relatives. These challenges can be related to sociocultural diversity, language barriers, and navigating the health care system [14]. Galiana-Gómez de Cádiz et al [15] highlighted the heavy burden of care and limited respite from caregiving responsibilities that immigrant informal caregivers often face. Informal caregivers, particularly those from racial or ethnic minority groups, often provide high-intensity care without help from formal caregivers, experience unmet needs, and may benefit from culturally sensitive programs and policies [16]. Immigrant informal caregivers often face challenges in accessing support, services, and resources because of the gendered nature of care work and their immigrant social locations [17]. Moreover, health care and community service providers face difficulties when providing culturally appropriate care to immigrant families. These obstacles include language and communication barriers, as well as differences in the understanding of disability [18]. In Sweden, the welfare system is reported to perceive asylum seekers as individuals whose grievances need not be given due consideration and who are seen as subject to deportation. This perception has unfortunate consequences, as it hinders their ability to access formal care systems and creates a significant demand for informal care [5]. Immigrant informal caregivers often face competing priorities and beliefs, feel out of control, and need education and culturally tailored support systems [19].

Digital technologies have become an increasingly important resource for supporting informal caregivers in traditional health care. Mobile health (mHealth) refers to the use of mobile devices such as smartphones, tablets, and wearable technology in health care. It encompasses various apps and services designed to improve health care delivery, patient monitoring, and access to health-related information through mobile technology. Most mHealth apps for caregivers provide educational resources and information to caregivers. Some telecare technology apps may also use remote monitoring tools and devices, such as wearable sensors and video cameras, to support caregivers in monitoring the well-being of their loved ones [20]. This technology has been shown to improve the quality of life of caregivers and care recipients and reduce the burden on the health care system. mHealth apps have also been used to provide social support for caregivers. For example, internet-based forums and social media groups have been created to connect caregivers, allowing them to share their experiences and offer support and advice to one another [21]. Care coordination platforms provide a centralized location for care information, allowing real-time communication and collaboration among caregivers, health care providers, and other support individuals [22,23]. By streamlining care coordination and reducing the administrative burden, care coordination platforms can help reduce caregiving’s overall stress and strain. This streamlining of care coordination can be particularly beneficial for caregivers responsible for coordinating care for multiple individuals or for juggling multiple care-related tasks. In addition, mobile apps provide various resources and tools to help manage caregiving duties such as scheduling and tracking medications, tracking care recipient health metrics, and managing finances. Mobile apps can help reduce the overall burden of caregiving by providing care-related information and resources at the caregiver’s fingertips. Mobile apps can also improve the quality of care, allowing caregivers to access information and resources to help make care decisions.

e-Coaching apps are a kind of mHealth app that provides users with coaching, support, and feedback through various methods, such as text-based conversations, audio and video calls, and interactive modules [24]. e-Coaching aims to help individuals improve their well-being, skills, and behaviors in a specific area or in general. e-Coaching apps can be used by individuals who want to achieve personal or professional goals or by organizations who want to support their employees. They offer the flexibility and convenience of being accessible anywhere and anytime, provided they have an internet connection. They also provide personalized support by adjusting the coaching content based on the user’s needs and progress [25].

In addition, e-coaching apps can be cost-effective compared with traditional coaching methods and can reach a larger audience. These apps have been used in health, wellness, and personal and professional development. In the health care field, e-coaching apps have been used to support individuals with chronic conditions such as diabetes or to provide mental health support to individuals struggling with stress and anxiety [26]. In informal caregiving, e-coaching apps can provide support, resources, and education to caregivers looking after older adult family members, friends, or loved ones [27]. These apps can help caregivers manage stress, provide caregiving tips, and connect them with support networks and resources. By leveraging technology, e-coaching apps can offer scalable and accessible support to caregivers, helping them care for their loved ones while also improving their well-being.

Designing mHealth apps with a persuasive design approach can be highly beneficial in encouraging positive behavior change and promoting health in immigrant informal caregivers. Designing mHealth apps using a persuasive design approach can be achieved through several approaches, including the Persuasive System Design Model (PSDM). The PSDM was introduced in 2009 to provide a comprehensive method for developing apps that use persuasive design principles. By using theories of social psychology, persuasive design aims to inform, persuade, and convince people to adopt new behaviors [12]. In health care, a persuasive design can be used to encourage healthy behavior and potentially prevent or manage illnesses. The PSDM offers a systematic approach to designing engaging and applicable interventions that involves analyzing major aspects of persuasive systems, understanding the context, and designing system qualities. However, the existing literature suggests that PSDM needs to be adapted to incorporate the use and user context [28]. In a recent study, the PSDM was adapted to the context of informal caregivers [27]. As part of this adaptation, the “self-monitoring” principle was reformulated, and 2 additional principles, “friendsourcing” and “peer mentoring,” were introduced to the PSDM. In this study, building upon this adapted model, we further extended it to incorporate the experiences of immigrant informal caregivers.

**Figure 1:**
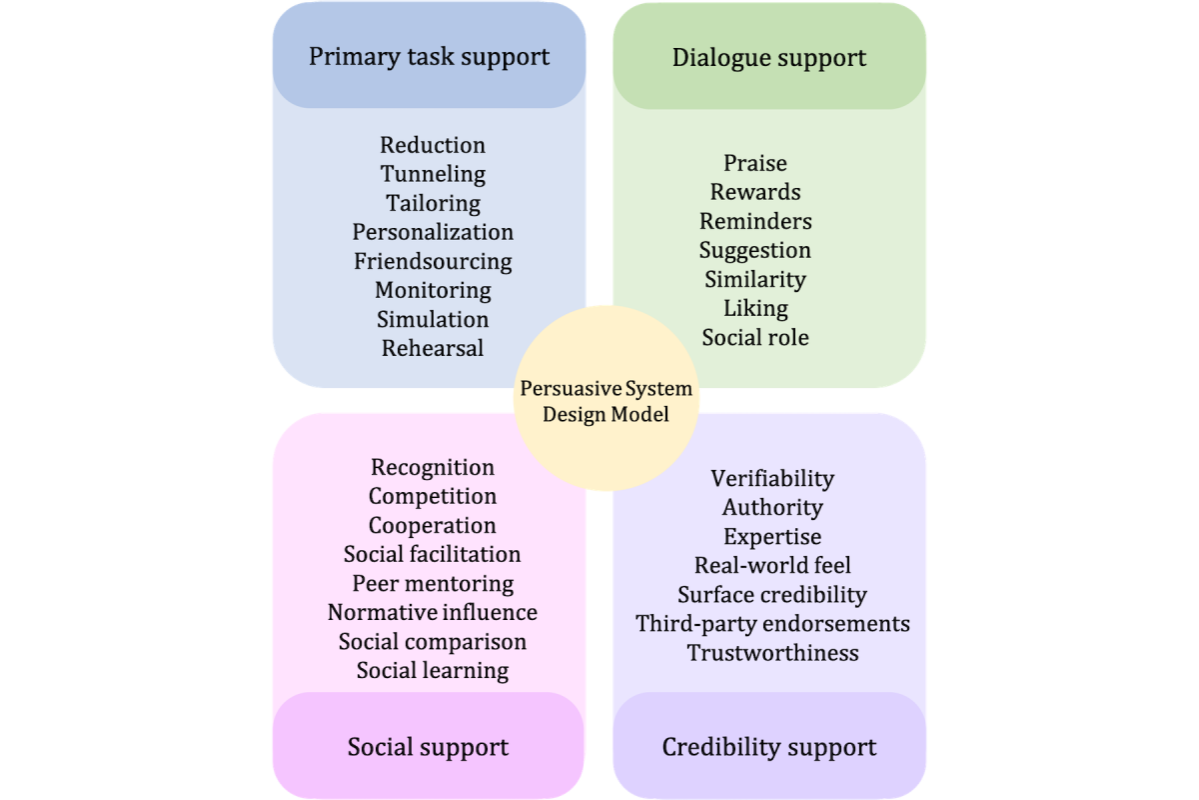
The adapted Persuasive System Design Model for informal caregiving.

The adapted PSDM [27] proposes 30 design principles or strategies that are categorized into 4 dimensions: primary task support, dialogue support, credibility support, and social support. These design principles are grouped into four dimensions, as shown in Figure 1 [25], which is an adaptation from the study by Oinas-Kukkonen and Harjumaa [29]: (1) primary task support that helps users perform their target behaviors; (2) dialogue support that uses design principles that motivate users through feedback and interaction with the app; (3) credibility support using techniques that make potentially prevent or manage illnesses look and feel trustworthy to users; and (4) social support, which uses techniques that leverage social influence.

### Research Objective and Questions

Despite the growing immigrant population in Sweden, there is limited knowledge about the challenges immigrant informal caregivers face [30]. These caregivers may face unique stressors such as language barriers, cultural differences, limited support networks, and financial difficulties [30]. Navigating the health care system, understanding medical instructions, and accessing information on the web can be particularly challenging, leading to increased stress and confusion in caring for their relatives. Immigrant informal caregivers may also struggle with cultural differences in their caregiving approach and navigating cultural norms and values in a new country [30]. These stressors combined with limited social networks and financial insecurity can lead to feelings of isolation and loneliness. The burden of caregiving can be further compounded by the need to balance work and caregiving responsibilities, making it a complex and challenging experience for immigrant informal caregivers [8]. Hence, this study explores the needs and challenges faced by immigrant informal caregivers and presents the design of a persuasive mobile e-coaching app. This paper addressed the following research question:

- How can a persuasive design approach be used to design a mobile e-coaching app, addressing the needs and challenges that immigrant informal caregivers face in Sweden?

The research question was divided into 3 subquestions:

- What are the needs and challenges immigrant informal caregivers in Sweden face?
- How do the persuasive design model principles address these needs and challenges?
- How can persuasive design principles be incorporated into the design of an effective mobile e-coaching app for immigrant informal caregivers in Sweden?

This study explores the use of the adapted PSDM in the designing of a mobile e-coaching app to pursue the aforementioned research questions. To begin this process, we conducted interviews with Swedish immigrant informal caregivers to discern their specific needs. We then formulated design recommendations rooted in adapted PSDM principles. These insights serve as the foundation for proposing modifications to the PSDM, tailored to the distinctive needs of immigrant informal caregivers in Sweden. Finally, we have presented the design of the mobile e-coaching app. Ultimately, our objective is to tackle the multifaceted needs and challenges faced by this group by advocating for the development of an e-coaching app that seamlessly integrates persuasive systems design principles.

## Methods

### Overview

Our research adopted a qualitative approach, beginning with the exploration of caregivers’ needs for a mobile e-coaching app. This research adopts an interpretive, evolutionary, and complementary ontological stance. Our research has been informed by a reflective and hermeneutic epistemological stance [31].

We gathered information on their caregiving experiences and context through semistructured interviews. This section outlines the participant recruitment process and data collection and analytical procedures. In addition, we describe the process of adapting the PSDM to the context of informal caregiving. We followed the COREQ (Consolidated Criteria for Reporting Qualitative Research) checklist (Multimedia Appendix 1) to report this study’s method and qualitative findings [32].

### Study Design

**Figure 2:**
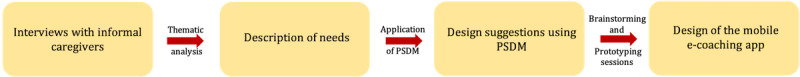
Study design. PSDM: Persuasive System Design Model.

Figure 2 illustrates a stepwise process of the study design described in this section.

#### Participants and Recruitment

The study was conducted in Uppsala, Sweden, over 8 months from April 2022. The participants were considered immigrants and included in the study if they and their parents were born outside of Sweden. They were recruited through the National Association of Relatives (Anhörigas Riksförbund), rehabilitation departments at municipalities, advertisements on Facebook groups, and public libraries in areas with a higher concentration of immigrants. We also recruited participants through Selmagruppen, a nonprofit association of immigrant women in Sweden that aims to help immigrant women integrate with and understand Swedish society.

Information leaflets were distributed in these caregiver associations, with links to the study’s page on the university’s web page. Caregivers were given the option to either use the web-based registration form to input their information or contact the research team via phone or email. In addition, the snowballing technique was used to reach out to certain caregivers, where they were contacted through referrals from the participants who had already been interviewed [33].

We received interest from 16 immigrant informal caregivers willing to participate in the study. However, only 13 of them could be interviewed. Three of these caregivers could not participate in the study because of other commitments, despite their initial interest. Caregivers who were interested in participating in the study were from the Uppsala and Stockholm regions. The demographic data of the participants and their care recipients are presented in Table 1. We used the American Medical Association’s age classification to determine the age of the caregivers and care recipients [34].

The research team did not have any relationship with the participants before the commencement of the study.

**Table 1:** Participants’ information^a^.

Age group of caregivers (years)	Sex of caregiver	Condition	Age group of care recipient (years)	Country
18-40	Female	Type 1 diabetes	65-90	Syria
18-40	Female	Autism	0-12	Morocco
18-40	Female	Autism	0-12	Iraq
18-40	Female	Autism and ADHD^b^	0-12	Yemen
18-40	Female	Autism and ADHD	0-12	Morocco
41-64	Female	ADHD and mental development delay	18-40	Egypt
18-40	Female	Glanzmann thrombasthenia	0-12	Syria
18-40	Female	Bone issues due to aging	65-90	Egypt
65-90	Male	Diabetes	65-90	Palestine
41-64	Female	Schizophrenia and hemiplegia	41-64	Syria
41-64	Female	Diabetes	41-64	Iran
18-40	Male	Diabetes	65-90	Syria
18-40	Female	Vestibular vertigo	41-64	Syria

^a^The care recipients are able to perform basic activities of daily living independently but are dependent on instrumental activities of daily living.

^b^ADHD: attention-deficit/hyperactivity disorder.

#### Procedure and Measures

A semistructured interview guide was used with open-ended questions [35] (Multimedia Appendix 2). SP, PÅ, and ÅC collaborated to create an interview guide approved by the Swedish Ethical Review Authority. The guide contained questions about informal caregiving, including caregiving tasks, experience, assistance from family and friends, respite care, and formal health care. It also included questions about the potential help and support needed by caregivers.

The interviews were conducted in Arabic by a research assistant employed at the university. The research assistant has had prior experience in conducting interviews for research projects. Before the interviews, SP had several mock interview sessions with the research assistant. Field notes were taken during the interviews by the research assistant, which were discussed with SP after the interviews to understand the participants better.

The interviews were conducted one-on-one and audio-recorded. After the interviews were completed, the research assistant transcribed and translated them into English for analysis. Although some informal caregivers preferred web-based videoconferencing [36] for convenience and flexibility, others preferred to do so in person at their homes. The interviews lasted between 60 and 75 minutes. The transcripts of the 13 interviews were reviewed by SP and ÅC, who thoroughly examined them and engaged in discussions regarding data saturation. On the basis of their analysis, it was concluded that data saturation had been sufficiently reached.

#### Interview Data Analysis

Data were pseudonymized and entered into qualitative data analysis software. Data were analyzed using the thematic analysis described by Braun and Clarke [35]. Following each interview, SP engaged in discussions with the research assistants to review the interview proceedings. During these discussions, SP took quick notes regarding the participant’s context as well as any noteworthy and interesting thoughts associated with it. SP reviewed the data repeatedly to gain familiarity with it while taking notes of ideas. To examine the needs and challenges encountered by immigrant informal caregivers, an initial set of codes was systematically developed by SP. Subsequently, SP and ÅC analyzed these codes and looked for broader themes, gathering the most relevant data for potential themes. The broader themes were then reviewed and refined by SP and ÅC to ensure their importance to the research question. Quotes were linked to relevant themes, and the most important themes were selected and defined to explore the needs of caregivers for a mobile e-coaching app. Finally, the themes along with the researchers’ interpretations and illustrative quotes have been described in the Results section. Data were stored and analyzed using the qualitative data analysis software MaxQDA. An inductive approach was used to explore caregivers’ needs for the e-coaching app.

#### Elicitation of Design Suggestions and Adaptation of the PSDM

The caregivers’ needs were mapped using the adapted PSDM. The description of the need was compared with the description of the persuasive design principles from the PSDM, and a design principle was chosen based on this match.

If the description of the needs did not map with any potential design principles from the PSDM, we described the design suggestion in detail and checked whether any design principles were close to it. Otherwise, we suggested a new design principle for the PSDM. Once we described the new design principle, we checked for any overlap with an existing design principle from the PSDM. Once no overlap was found, we formally added it as a design principle in the extended model.

### Prototype Design

**Figure 3:**
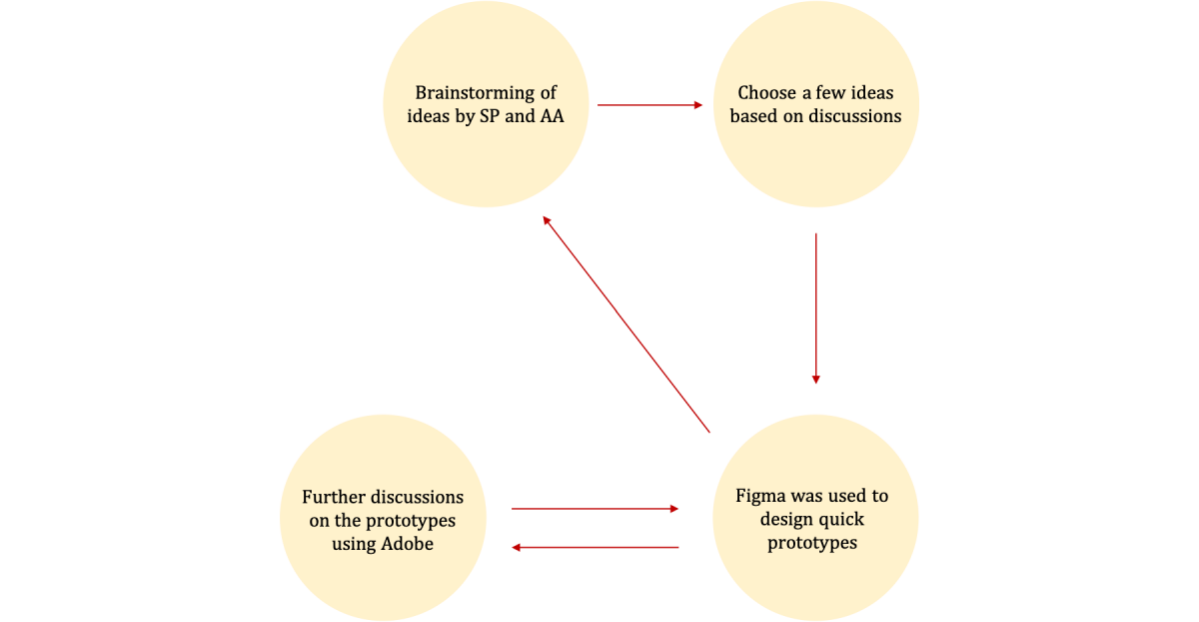
Prototype process.

During the brainstorming sessions, SP and AA generated multiple ideas for the prototype, as illustrated in Figure 3. Careful attention was paid to the description of the informal caregivers’ needs and adherence to the adopted persuasive system design principles to ensure that the resulting prototype would be responsive to users’ requirements. To visualize and discuss the initial ideas, Figma, a prototyping tool [37], was used. However, as the prototype progressed, Adobe Illustrator was used to create esthetically appealing prototypes [38]. In the Results section, the main screenshots of the prototype that address the needs of informal caregivers are presented. These visuals demonstrate how the prototype meets specific requirements and showcase its features and user interfaces.

### Ethical Considerations

The Swedish Ethical Review Authority approved this research project (Dnr: 2021-03656), which was conducted with verbal and written information provided to participants. Written informed consent was obtained before the interviews. At least 2 weeks separated participants’ receipt of information about the study and their consent to participate—enough time for them to consider their involvement. No relationship was found between the authors and the informal caregivers. Some participants experienced emotional distress or discomfort during this process. If this occurred, the interview was stopped to contact the informal caregivers’ association for assistance.

## Results

### Overview

**Figure 4:**
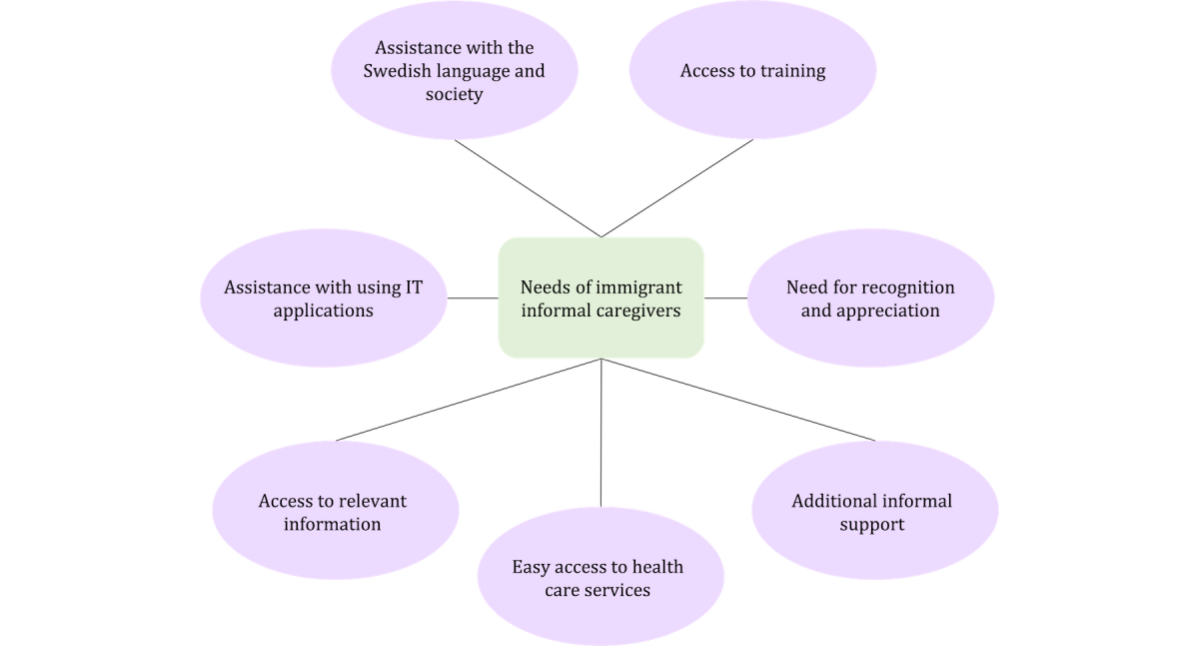
Needs of immigrant informal caregivers.

In this section, we reveal the findings from our empirical data and answer the research questions related to the design of the mobile e-coaching app. The first research question focused on identifying caregivers’ needs through semistructured interviews. In this section, we describe the needs of immigrant informal caregivers. Figure 4 summarizes these needs. The second research question pertains to the design suggestions that can address the needs of immigrant informal caregivers identified in the first research question. We proposed design suggestions using the adapted PSDM for each requirement. These suggestions are based on our interpretation of the needs and understanding of the persuasive system design principles. Finally, to answer the third research question, we present the prototype and a visual representation of the proposed design.

### Description of Needs

#### Access to Training

Most caregivers reported that they had taken some formative training courses on the illness, especially those who cared for a patient with autism. They reported that these intensive courses were aimed at teaching them to provide care to their care recipients, especially in the early years after diagnosis. In addition, some caregivers also expressed that they would like to have training on what to expect a few years later as the condition or the age of the care recipient or caregiver advances. They felt that such training could help them be better prepared in their caregiving process.

Some other caregivers (especially those caring for patients without autism) were not aware of such training courses but emphasized that access to such courses early on would certainly be helpful and enable them to be better prepared. Some caregivers felt that the formal health care system, at different stages, needs to anticipate challenges that caregivers may face and design courses that could help them:

I went to [“rehabilitation for autism”] to take care of autistic children and took training courses once every two weeks, meaning twice a month. I attended training courses for six weeks to learn more about autism and how to deal with my child to improve and develop.

Morocco, aged 37 years, son with attention-deficit/hyperactivity disorder (ADHD) and autism

At the moment, I need to cover his free hours and also I mean, I also want training courses that teach me how to deal with him as a teenager. /.../ When he turns eighteen how his relationship with the opposite sex will be and how I will explain to him what it means to deal with... (she is referring to girls with her gestures).

Yemen, female, aged 44 years, son with ADHD and autism

#### Assistance With the Swedish Language and Society

Most participant caregivers stated that they needed help with understanding the Swedish language. As most of the information and services from the counties are provided in Swedish, it becomes even more difficult for immigrant informal caregivers to find and understand these information and services. This creates an additional challenge for these caregivers who may already be dealing with the demands of caregiving while adjusting to a new country and culture. They feel that if they could learn the language, they would be able to search for information related to caregiving, which would ultimately help them better serve their relatives.

In addition, they feel that Swedish culture is quite different from their native culture in many aspects, especially when interacting with government agencies and traditional health care systems. Differences in communication styles, values, and expectations may lead to misunderstandings or challenges in navigating these systems and situations. Cultural differences in communication may also lead to difficulties in understanding and following medical instructions or in communicating with medical officials. Hence, they expressed that understanding the nuances of living in a society so different from their native society is challenging, especially when they are also caregivers:

I wish there were a program that would help me learn the language so that I could care and learn at the same time. To learn how to search for anything I want for my son and to learn the language to complete my studies in the same field because I chose to work in the same field and help people with special needs.

Egypt, female, aged 48 years, son with ADHD and mental development delay

#### Assistance With Using IT Applications

Most immigrant informal caregivers expressed that they had limitations in accessing web-based content and using web-based applications. Their internet use was limited to Google and YouTube, and they felt that this was insufficient, as most information and services in Sweden were hosted on the internet. They highlighted the need for assistance in using such IT applications. This assistance may include guidance on how to install apps from app stores and how to use them effectively. For example, caregivers may need assistance using health care apps to book appointments or access medical records.

Moreover, some of them have not used IT systems much. Hence, they would need support throughout the process of using an IT application, from the initial installation to achieving a desired outcome. This may involve technical support for troubleshooting and resolving any issues that arise while using the app. Most caregivers reported that they do not have experience using IT applications and would need help while using them to accomplish their goals:

I research diabetes and others, [using] my computer. [I] only use Google and not any other page. I honestly haven’t tried any mobile applications [apps] or websites. I have no experience in it and have not tried to learn it. It takes time and I don’t have so much time to learn this.

Syria, male, aged 30-40 years, father with diabetes

#### Need for Recognition and Appreciation

Most immigrant informal caregivers felt that they lacked a general acknowledgment of their efforts from immediate or extended family members. According to the interviewed female caregivers, they felt this was due to the hierarchical man-woman relationships in their culture. They felt that some degree of understanding, recognition, and appreciation would go a long way to boost their morale and help them continue to provide care to their relatives. Some also felt that such motivation could be good for their mental health, as they spend a lot of time wondering if they are doing things right by their relatives.

Some caregivers spoke about the sacrifices they had to make to care for their relatives, for example, missing out on certain job opportunities and ignoring their physical limitations. They felt that an acknowledgment of them from the care recipient and their extended family members could instill a renewed sense of purpose and motivation. They felt that this could also strengthen their familial bonds, especially in the extended family:

He is a lazy man at home! Because of, as I said, the men who come from the Middle East, they are like served in the home, and he has been like that from the beginning [this statement is the participant’s own views and is not a generalized statement reflecting the Middle East]... I have to fix, clean, do a lot. Not just like putting out breakfast or food, it’s cleaning, washing up, and laundry. So maybe I will prepare lunch for like half past one, twelve-thirty. And also that- he complains a lot. /... / he will sit and eat lunch with medication; he has special medications to take with food. He goes to bed, the TV is on, the laptop, mobile phone, Facebook, and so. But now it has become a little like I’m thinking about myself. I myself need some space, and that’s what helps me, I have a lot of girlfriends who tell me I am doing good. It’s like… you need that sometimes to... It helps to keep going.

Iran, female, aged 63 years, husband with type 1 diabetes

#### Access to Relevant Information

Most immigrant informal caregivers stated that it would be helpful to access relevant information in their native language. They have had difficulty accessing information in languages other than their own, particularly Swedish, when researching topics such as autism. They mentioned that they often read books about topics such as autism, hyperactivity, and disability but wished this information was more readily available in other languages.

They were also worried about the trustworthiness of the information. They expressed concern about the reliability of the information they found on the internet and read multiple sources to confirm its accuracy, as they fear encountering false or incorrect information. They stated that most of the time they spent was to confirm the accuracy of the information by visiting multiple websites or talking to other caregivers:

And it’s also another thing that I don’t know how it is to have information in all other languages. For example, if I want to find out information about autism it’s difficult. Sometimes I read books on my own about hyperactivity or disability, in general, but I wish this information were available in other languages in Sweden, for example, it would have made many things easier… I write the question, and when I open it, several sites appear to me. Then I read the first three until I find that the speech applies to all of them. Because sometimes I’m afraid to take false information. Or wrong answers, for example. But I still can’t tell if the information is 100% true.

Egypt, female, aged 48 years, son with ADHD and mental development delay

#### Additional Informal Support

Most immigrant informal caregivers felt alone in their caregiving journey in terms of feeling that they were the only ones providing care for their care recipients. This was particularly felt by female caregivers who balanced caregiving responsibilities with other activities such as studying or working. As a result, many female caregivers emphasized the need for support from other family members or friends when they have other activities to focus on, such as their education or a job.

The caregivers interviewed stressed the need for a joint coaching session with family members and health care providers to support the primary caregiver. This could involve bringing together family members, health care providers, and the primary caregiver to discuss the needs and challenges associated with caregiving. By working together, these stakeholders could develop a plan to support the primary caregiver and ensure that their relative’s needs are met:

[If my friends have to look after him] The person who stays with him must be able to understand his character. Someone who [can] expect what my child can do. For example, if he is sitting at the table, I know he will fall and injure himself, and I know how impulsive he is. And he can cause a problem because they will say that he is a normal child, and he can cause a problem.

Morocco, female, aged 34 years, son with autism and ADHD

#### Easy Access to Health Care Services

Most immigrant informal caregivers experienced difficulty in accessing doctors or having medical assistance on time. They felt that they had to wait for long hours and would only be able to see a nurse for a short period, leading to another waiting time for a physician’s appointment. This made them feel that their care recipient’s health was not taken seriously.

In addition, some caregivers struggled to understand the Swedish health care and insurance system, making it difficult to navigate the system and access appropriate services. This lack of understanding may be compounded by comparisons with health care systems in their home countries, which may have been more familiar and relevant to them. There were frequent comparisons to the health care system, the benefits or aid they received back home, and how that was more relevant. Immigrant informal caregivers stressed that by providing timely and efficient health care services, caregivers may be better able to manage their care recipient’s health needs and feel more supported in their caregiving role:

I would like something that can help get easy access to the doctors and the medical assistance because it is very hard to get that on time here in Sweden. It would also be nice if there is a file with all patient information and that is easy to access or a link or a website that helps the caregiver know what to do in an emergency.

Morocco, female, aged 37 years, son with autism and ADHD

### Mobile e-Coaching App Design

#### Overview

**Figure 5:**
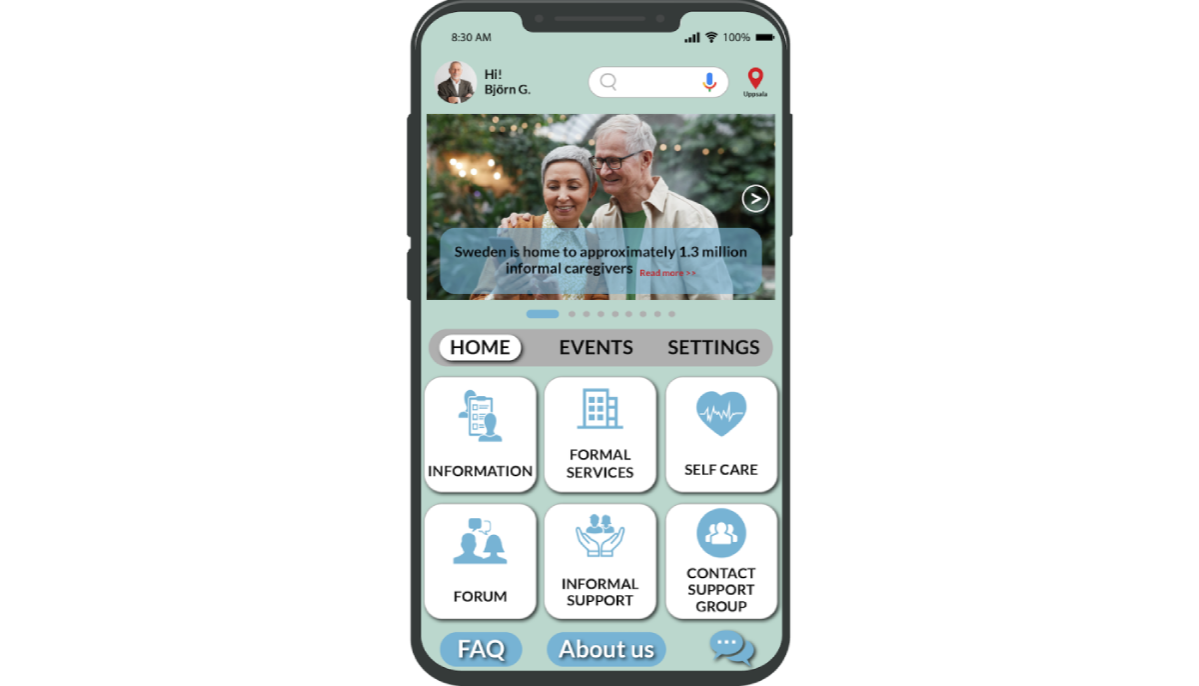
Home page of the app.

In this subsection, we first present the design suggestions for the mobile e-coaching app using the adapted PSDM. Finally, we present an overview of the various functions of the mobile e-coaching app, outlining how each function is tailored to address the caregivers’ needs. The main page of the app encompasses a range of functions, including “information,” “formal services,” “self-care,” “forum,” “informal support,” and “contact support group,” as depicted in Figure 5. We also delve into the practical implementations, demonstrating how caregivers’ needs and PSDM design suggestions have been practically integrated into the aforementioned functions.

#### Access to Training

The “tailoring” design principle of PSDM can be applied to address this specific need, which is to provide caregivers with information on available training courses on the internet that are relevant to the illness and age of the patient, the relationship with the patient, the caregiver’s age, and the caregiving stage that they are in. The app can personalize the information presented to the caregiver based on their location, care recipient’s condition, caregiver’s age, and stage of the caregiving process, considering their unique situation.

**Figure 6:**
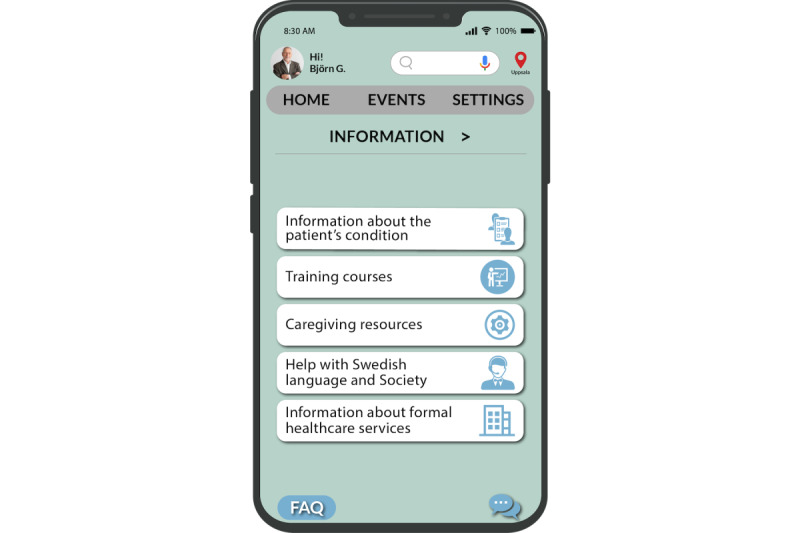
Information page.

**Figure 7:**
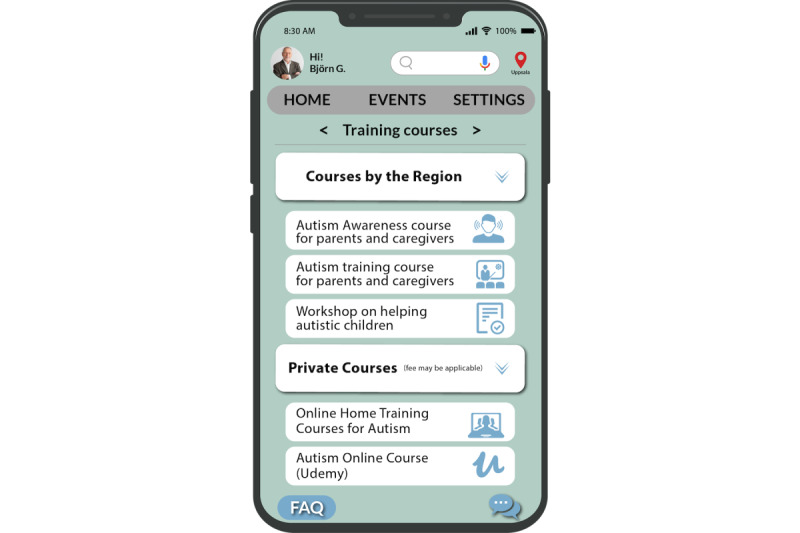
Training courses.

To further enhance the app’s effectiveness, it is essential to anticipate caregivers’ problems and provide them with relevant training sessions that will equip them to address future challenges. By doing so, caregivers can be adequately prepared to handle different scenarios in their caregiving journey, such as managing the patient’s changing health conditions or coping with emotional and mental stress (Figures 5-7).

On the main page of the mobile e-coaching app, we introduced an “Information” option (Figure 5). This option provides access to all relevant information about caregiving that can be useful for caregivers (as depicted in Figure 6). Within this “Information” page, among other options, caregivers can view information on the training courses available to them. We present information about 2 distinct types of courses: one facilitated by the state and the other by private organizations (as depicted in Figure 7). Here the caregiver is providing care for “autism” and hence can readily access courses tailored to their needs, such as “Autism Awareness for Parents and Caregivers.” This is an implementation of the “tailoring” design principle.

#### Assistance With the Swedish Language and Society

The PSDM’s “suggestion” design principle can significantly enhance the effectiveness of an app by providing caregivers with various options and resources to assist them in their tasks. To this effect, the app can offer a variety of resources, such as links to useful websites and books, tips and advice, and a glossary of Swedish medical terms and their meanings. This can help caregivers stay informed and be equipped to effectively manage their caregiving responsibilities.

Furthermore, the app can also incorporate the “social learning” design principle, which can facilitate interaction and community building between Swedish native and immigrant informal caregivers. This can be achieved by providing a web-based platform or space where caregivers can connect, share their experiences and knowledge, and learn from each other. This feature can be especially beneficial for immigrant informal caregivers who are unfamiliar with the Swedish language and culture. By engaging in conversations with native Swedish caregivers, they can gain insight and knowledge about the country’s society and language, making it easier for them to integrate into the community. The provision of this web-based platform or space in the app can also provide a platform for caregivers to connect and network. Caregiving can be an isolating experience and having a community of like-minded individuals can be a resource for support and advice.

**Figure 8:**
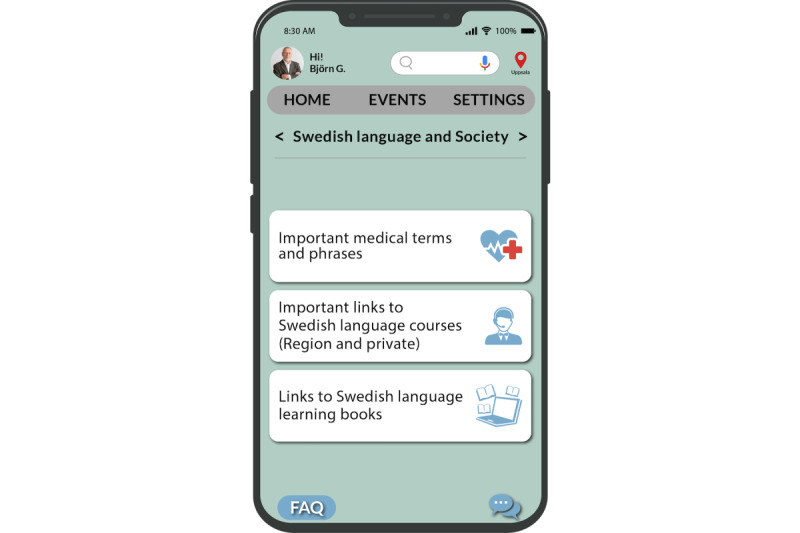
Swedish language page.

**Figure 9:**
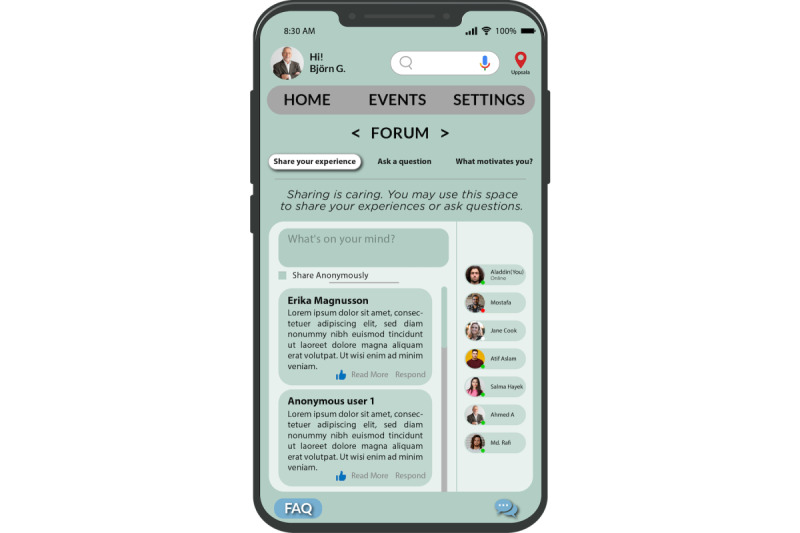
Forum page.

We designed a dedicated section to assist the caregivers in the Swedish language and society (Figure 8). They can access this section from the “Information” page (Figure 6). This section encompasses resources that include medical terms, information on Swedish language courses, and an array of learning materials. Notably, the app also includes an internet-based “Forum” (depicted in Figure 9), fostering connections among caregivers, especially with Swedish caregivers. This platform provides an opportunity for collaboration, networking, and cultural exchange and facilitates a smoother integration process.

#### Assistance With Using IT Applications

Immigrant caregivers have shown a limited understanding of using IT applications and therefore need assistance for a complete user journey, from installing the app from the App Store to obtaining some potential benefits from the app. Such support could include web-based tutorials, help files, and technical assistance. By providing such assistance, immigrant informal caregivers may be better able to access important services and information on the internet, ultimately assisting them in providing care and support to their relatives.

To assist caregivers better in understanding and using the app, an advanced level of support can be provided. Here, we introduce a new design principle to the PSDM called “facilitating conditions.” It provides support structures that help users effectively navigate and use the system. The implementation of this design principle aims at making the app easier to use.

This feature can be especially beneficial for caregivers with limited technology experience or who require additional assistance in navigating the app’s features. The advanced level of support can also aid caregivers in understanding the app’s functionalities, ensuring that they can effectively use the system to manage their caregiving responsibilities.

**Figure 10:**
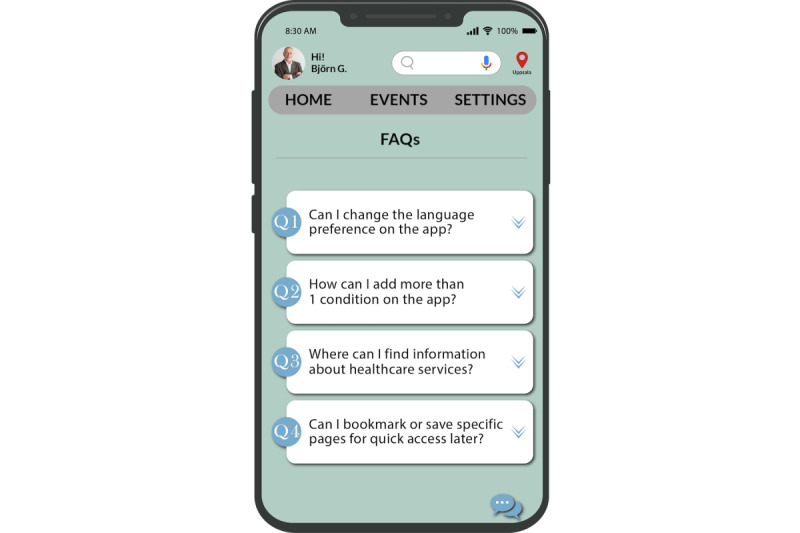
Frequently asked question page.

**Figure 11:**
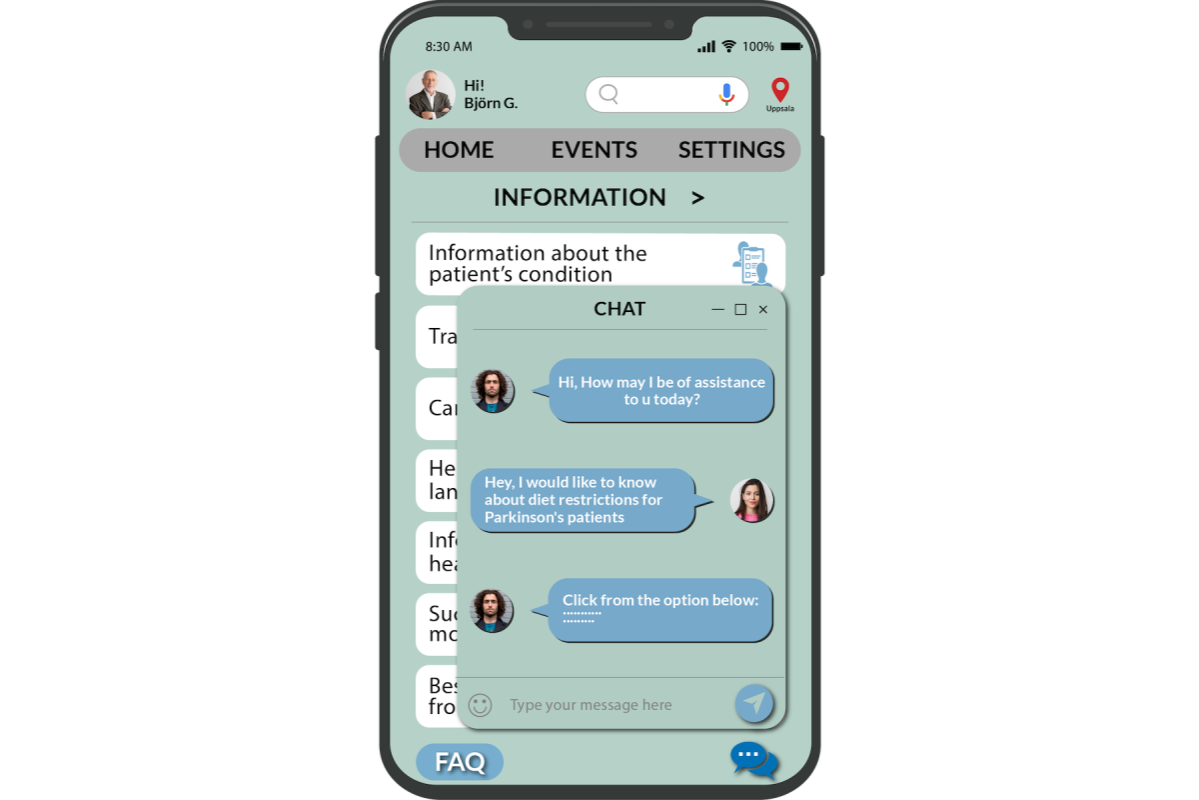
Chat page.

Within the app, we implemented 2 particular functions: frequently asked question (FAQ) and “menu-based” or “decision tree” chat. These functions were intentionally designed for optimal accessibility and usability, remaining accessible from any page or section of the app (as illustrated in Figures 10 and 11). The FAQ page serves as a repository of general information about the app’s functionalities. For instance, users can find guidance on tasks, such as adjusting language preferences within the app. The menu-based chat provides users with helpful instructions and assistance based on predefined options. These 2 functionalities combine to create a user-friendly support system, ensuring that users can easily navigate the app and receive the help they need in a manner that suits their preferences.

#### Need for Recognition and Appreciation

None of the 30 PSDM principles can be used to address this need of recognition and appreciation directly. Hence, we introduced a new design principle called “verbal encouragement.” Its aim is to provide motivational messages and encouragement to continue in the users’ tasks. Verbal encouragement can be implemented in the app by providing caregivers with inspirational and motivational experiences, best practices, or relevant tips from other caregivers. This could serve as a source of inspiration and validation for caregivers who feel overwhelmed or unappreciated (Figure 9).

In this e-coaching app, we developed a “Forum” space that offers caregivers a platform to share their experiences and situations. This forum, as illustrated in Figure 9, is not moderated and provides caregivers with a platform to share their experiences, ask questions, or simply vent their frustration. Caregivers can provide tips and advice based on individual caregivers’ specific needs and challenges. Caregivers can also share anecdotes about what motivates them in their caregiving journey. They can use the “What motivates you?” tab. By sharing their stories and engaging in discussions, caregivers can gain a sense of validation and acknowledgment, which is an implementation of the “verbal encouragement” design principle. Ultimately, this may contribute positively toward their well-being and foster a sense of community through the app.

#### Access to Relevant Information

In the mobile e-coaching app, all information presented to caregivers will be sourced from credible and official web pages to ensure its accuracy and trustworthiness. Through this, the “trustworthiness” design principle of the PSDM will be implemented here to instill confidence in caregivers regarding the reliability of the information provided. The system will display the information source (as depicted in Figure 7), providing caregivers with transparency regarding where the information comes from, further enhancing the app’s trustworthiness.

To make the information more relatable and engaging for caregivers, the “similarity” design principle of the PSDM will also be used. This principle involves presenting information in a way that imitates the user in a specific manner, creating a more personalized experience. Caregivers will be presented with information in their language and using texts and pictures that they can identify and relate to. By presenting relatable information, caregivers are more likely to engage with the app and feel more comfortable using it.

#### Additional Informal Support

In this e-coaching app, we provide a provision to schedule joint coaching sessions for family members and close friends with the health care professional. In the app, caregivers can also provide a quick summary of information that family and friends can use to pitch in. Caregivers can present a summary of the care recipient’s condition and any notes the caregiver has entered related to the care recipient’s illness, such as a quick fact sheet, on how to deal with unexpected situations in their absence.

**Figure 12:**
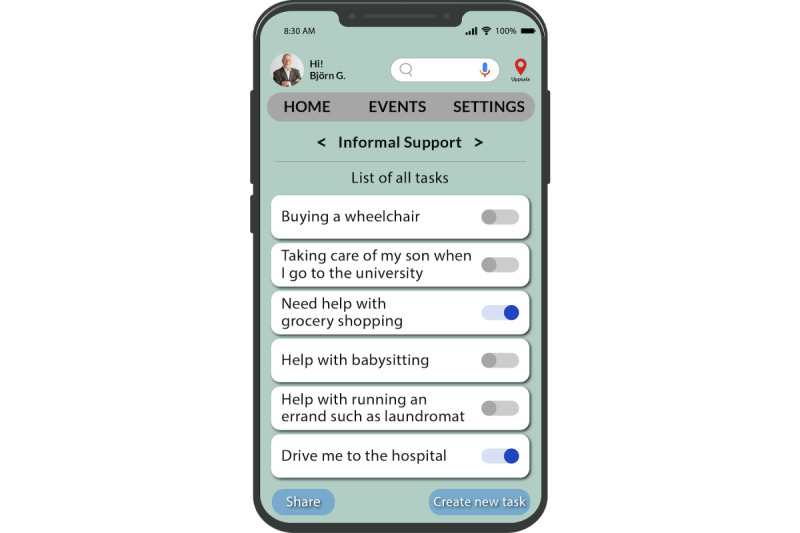
All tasks page.

**Figure 13:**
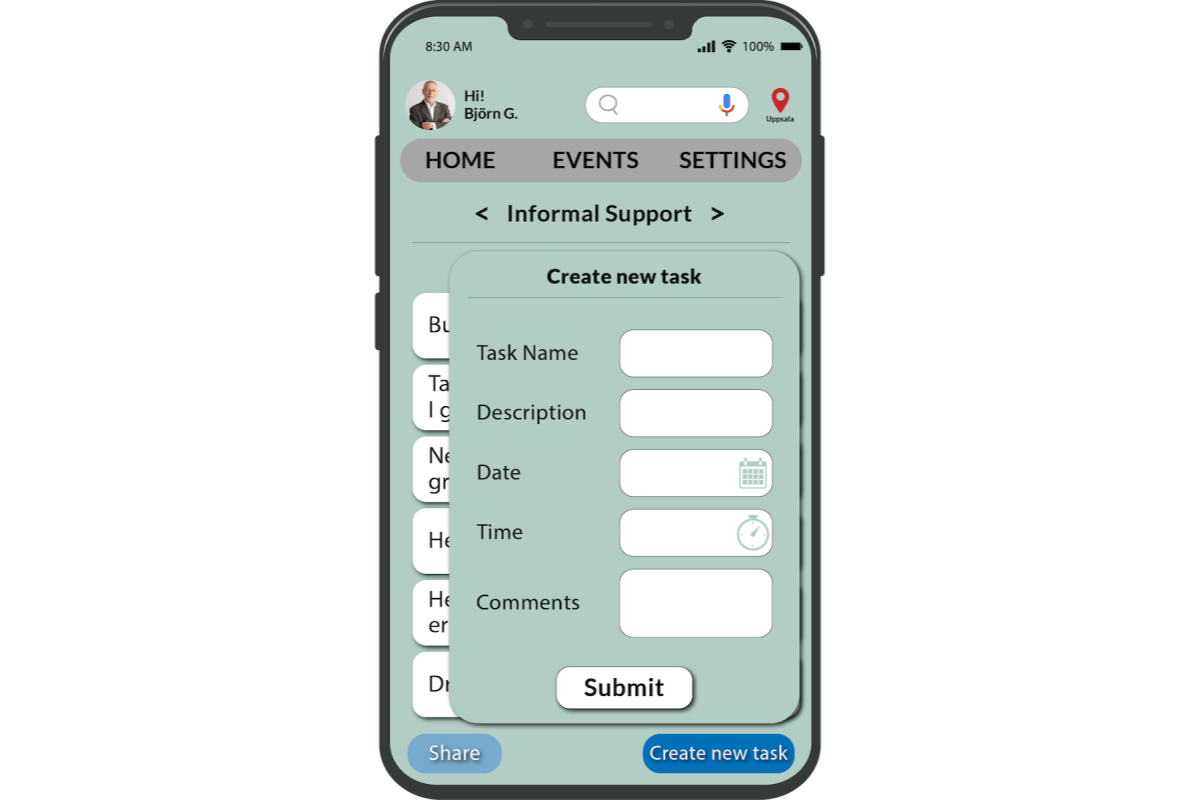
New task page.

**Figure 14:**
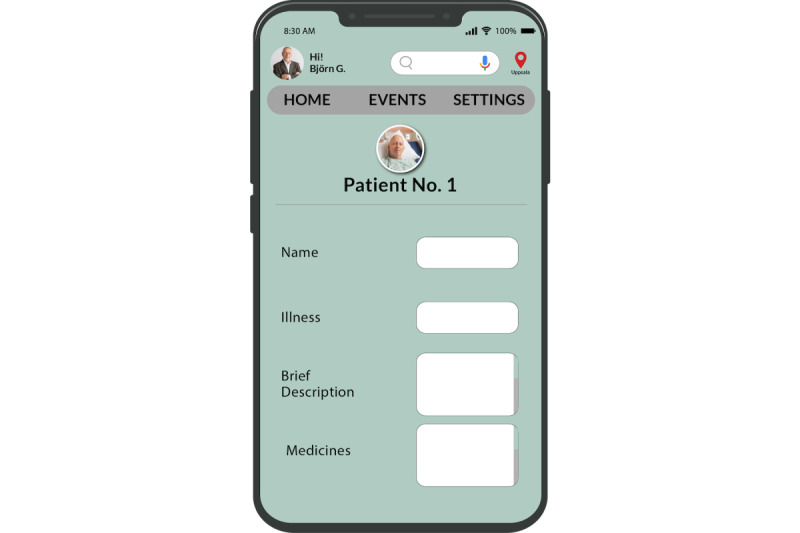
Patient information page.

We have also used the “Friendsourcing” principle [27] that motivates individuals such as family and friends in the caregiver’s community or care volunteers to provide informal support to them. Caregivers can create a list of tasks in their account, and these care volunteers can choose the tasks they can assist with. This feature aims to unite caregivers and their care volunteers on a single platform to work together on tasks. On the basis of their previous contributions, the app can also recommend tasks to family and friends (Figures 12-14).

**Figure 15:**
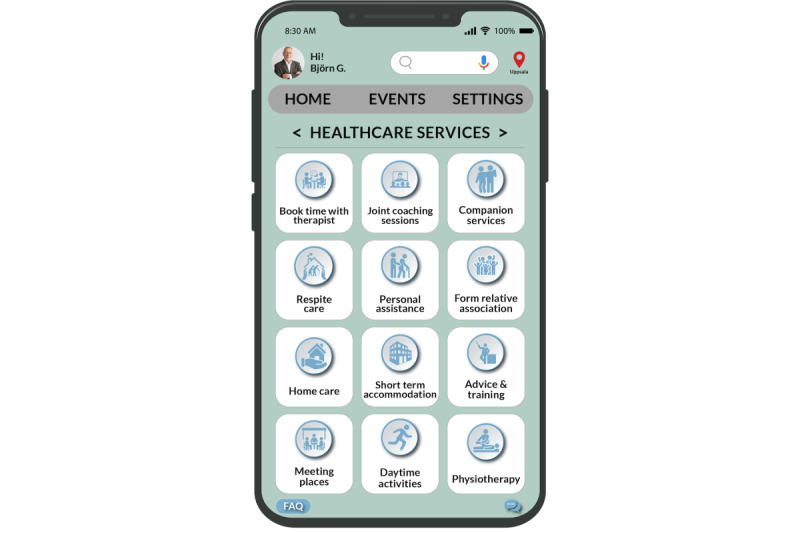
Health care services page.

In this e-coaching app, we provide a provision to schedule joint coaching sessions for family members and close friends with the health care professional on the health care services page (Figure 15). Caregivers can choose to offer a summary of the care recipient’s condition (such as a quick fact sheet) to their care volunteers. In addition, they can provide notes containing essential and pertinent details about caring for the individual and instructions on handling unforeseen situations when they are not around (Figure 14).

Caregivers also have the provision to share noncaregiving tasks with care volunteers when they need additional support (Figure 13). The tasks that the caregivers need repeated help with are stored in the app and provided as suggestions to begin with, as depicted in Figure 12. To access these lists of tasks, a caregiver’s friends or family would need to log in to the app as a care volunteer through an invitation from the caregiver.

#### Easy Access to Health Care Services

One way to apply the PSDM to address this need is to use the principle of “tailoring” to provide caregivers with personalized information about health care services and how to access them on the “Information” page. This could be done by gathering information about the caregiver’s location, the care recipient’s medical needs, and preferred language and then providing customized information about relevant health care services that meet those criteria.

Another persuasive system design principle that could address this need is “social learning.” Caregivers could be provided with opportunities to connect with other caregivers through the e-coaching app on the “Forum” page, allowing them to share information and support each other as they navigate the health care system. This could help alleviate some of the stress and frustration associated with accessing health care services and could provide caregivers with a sense of community and belonging.

To ensure easy access to information about various health care services, we introduced a dedicated page titled “Healthcare Services” (as depicted in Figure 15). On this page, we offer information tailored to parameters such as care recipient’s needs, caregiver’s location, and care recipient’s age. For instance, caregivers can find details on tasks, such as scheduling appointments with therapists, arranging personal assistance, and acquiring home care services.

### Adaptation of the PSDM

**Figure 16:**
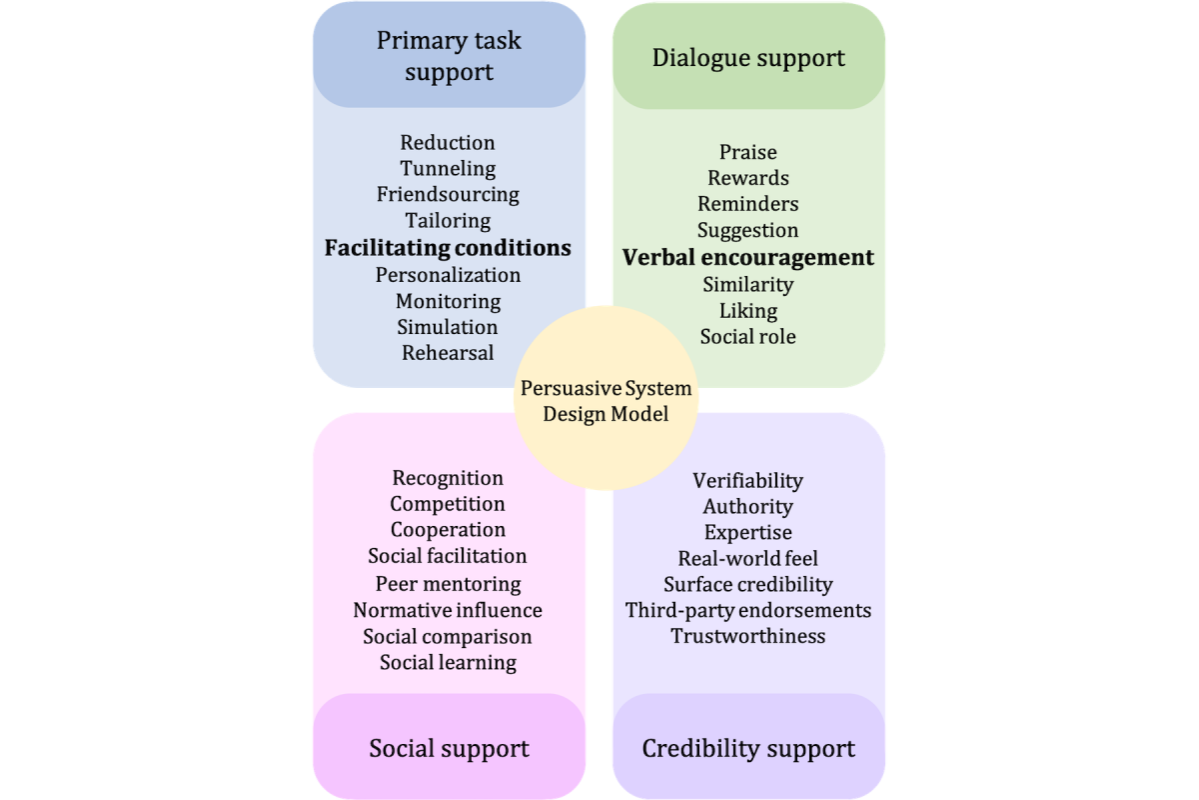
Persuasive System Design Model for informal caregiving (PSD-I).

Some of the needs, such as *assistance with using IT applications* and *the need for recognition and appreciation*, could not be mapped to any specific existing design principles in the PSD model. Hence, we extended the PSDM for informal caregivers, as illustrated in Figure 16. For the need *assistance with IT applications*, caregivers wanted to be able to receive assistance while using the IT application. This design principle of persuading the user by providing a support structure or help in the IT application has not been addressed in the existing PSDM. Hence, we introduced a design principle called “facilitating conditions.” In “facilitating conditions,” users can find help files or take the assistance of the menu-based chat to have help while using the IT application.

In addition, for the *need for recognition and appreciation*, caregivers felt the need to be recognized and appreciated for the work they were doing. This kind of persuasion technique is not addressed in the existing PSDM; hence, we introduce the design principle of “verbal encouragement.”

## Discussion

### Principal Findings

This study aimed to identify the needs of immigrant informal caregivers in Sweden and propose design suggestions and a prototype for a mobile e-coaching app to support these caregivers. These informal caregivers provide care for various medical conditions, such as autism, diabetes, ADHD; therefore, the aim is to discuss the common needs of caregivers. Hence, we have designed a generic mobile e-coaching app that may limit the specificity of information and coaching that can be provided compared with an e-coaching app focused on a specific condition. Through various channels, such as the National Association of Relatives, rehabilitation departments at the municipalities, and immigrant groups, we recruited 13 immigrant informal caregivers. These immigrant informal caregivers were mostly women and belonged to the age of 18 to 40 years. Most of the immigrant caregivers belonged to the Middle Eastern region, whereas some were from North African countries. However, all of them spoke Arabic. On the basis of the qualitative findings, 7 needs were identified: training access, language and societal assistance, IT application assistance, recognition and appreciation, access to relevant information, informal support, and easy access to health care services. The adapted PSDM [27] was used to propose design suggestions to address these needs. Although the PSDM is a useful tool for designing interventions, this study found that it needs to be adapted to fit the context of immigrant informal caregivers. As a result, the researchers added new design principles, namely facilitating conditions and verbal encouragement, to the PSDM, specifically for informal caregiving, PSD-I. The PSD-I model proposed in this study can guide information system researchers and designers in developing inclusive IT-based support interventions for informal caregivers. We do not claim to generalize statistically to “all immigrant caregivers” but rather apply our findings to theory [39]. Further studies are needed to generalize this result to a wider population.

This study also underscores the significance of early involvement and engagement of the ultimate end users, the caregivers, in the design and development process of mHealth apps. IT systems have shown considerable potential as efficient and cost-effective tools for delivering health care in the home environment. Nevertheless, the uncertainty surrounding their acceptance and adoption by end users calls for further investigation into the procedures and processes essential for the successful adoption of these systems [40-42]. Recognizing the well-being of end users and the genuine involvement of users in designing mHealth apps have emerged as pivotal factors in ensuring technology acceptance [42]. Within the scope of this study, we explored the circumstances and contexts of immigrant caregivers, valuing their unique needs and perspectives. This understanding serves as the foundation for designing and developing a mobile e-coaching app that is finely attuned to support these caregivers in their caregiving roles. As it aligns with existing research, we emphasize that gaining an understanding of caregivers and their needs is beneficial at the beginning of the design process. This contributes to the field of information system design.

Our findings indicate that immigrant informal caregivers value and need training for caregiving activities, which is in line with past research that has highlighted the importance of providing training and support to caregivers. For instance, a review by Sörensen et al [43] found that caregiver interventions, including psychoeducation and skills training, were associated with improved well-being, patient outcomes, and health care use. Similarly, a meta-analysis by Cheng et al [44] emphasized that caregiver interventions, including education and training, improve caregiver knowledge, self-efficacy, and mental health outcomes.

Moreover, studies have shown that tailored interventions may be more effective than generic interventions in improving caregiver outcomes than generic interventions [45]. Therefore, the application of the “tailoring” design principle suggested aligns with previous research, highlighting the importance of providing tailored interventions and support to caregivers. By providing personalized information and training sessions that address the unique needs and challenges of individual caregivers, the proposed app can improve caregiver outcomes and enhance preparedness in their caregiving journey.

On the basis of our findings, immigrant informal caregivers need assistance with the Swedish language and society. In countries with multicultural populations, extensive evidence indicates the importance of cultural and linguistic competence in health care. However, in Sweden, despite the growing multiculturality, only a limited number of studies have specifically addressed the challenges related to cross-cultural care [46]. Immigrant female family caregivers are known to avoid certain formal services for a variety of reasons, including the lack of cultural sensitivity and language issues [47]. On the other hand, health care providers also are often known to encounter language and communication issues when providing care to immigrant informal caregivers [18]. Moreover, immigrant informal caregivers face cultural differences between their native culture and Swedish culture, which further complicates their caregiving experiences. Interacting with government agencies and health care systems may be particularly challenging owing to differences in communication styles, values, and expectations [48]. These cultural differences can lead to misunderstandings, difficulties in following medical instructions, and ineffective communication with medical officials [49]. Immigrant informal caregivers highlighted the need to understand the nuances of living in a society that is different from their own, especially while fulfilling their caregiving responsibilities [50]. The findings suggest that health care and social service providers need to be aware of the unique challenges faced by immigrant informal caregivers and develop strategies to address their needs. Language interpretation services can empower immigrant informal caregivers to access information and services more effectively [18]. Culturally sensitive and inclusive approaches are essential for health care providers to bridge the communication and cultural gaps, ensuring effective collaboration and understanding between immigrant informal caregivers and health care professionals [51]. To improve the experiences of immigrant informal caregivers, the literature suggests the importance of tailored support services that consider the specific needs and challenges faced by this population [52]. Collaborative efforts between health care providers, community organizations, and policy makers are necessary to develop policies and programs that address the unique needs of immigrant informal caregivers and ensure equitable access to health care and social services [47]. In the e-coaching app, important links to web pages to learn the language and culture can be useful. This is addressed using the design principle of “suggestion.”

Our study and other related research also highlight that immigrant informal caregivers have limited knowledge and experience and little support for IT applications, and they need assistance in installing and effectively using them [53,54]. Their internet use is mainly limited to Google and YouTube, which they feel is insufficient as most services in Sweden are on the internet [55]. Limited experience with mHealth apps necessitates support throughout the process [54]. Immigrant informal caregivers’ lack of familiarity with IT applications underscores the significance of assisting in helping them accomplish their goals. By facilitating a complete user journey from installing apps from the App Store, caregivers can better access essential services and information through the use of the e-coaching app. In the existing PSDM, no principle specifically addresses the provision of support structures [29]. Therefore, we extended the PSDM by adding a design principle, “facilitating conditions.” Support functions such as FAQs, help files, and menu-based chats can help enhance the app’s persuasiveness.

This study also highlighted that immigrant informal caregivers, especially women, often lack acknowledgment from family members, impacting their morale and mental health. Recognition and appreciation are crucial for their motivation and well-being as they navigate the challenges of caregiving [6]. Therefore, we introduced the “verbal encouragement” principle to address caregivers’ needs, which was not present in the existing PSDM. It provides inspirational experiences, best practices, and tips for other caregivers. Caregiving might be emotionally and mentally draining, and the added stress of feeling unappreciated can exacerbate mental health issues [56,57]. Recognizing and appreciating caregivers can serve as a great source of motivation, especially when they face the demands and challenges of caregiving [6,58]. It is also perceived as beneficial for their mental well-being as they often question whether they adequately meet the needs of their relatives. In our app, we propose inspirational content, best practices, and a forum for caregivers to share their experiences and seek advice, addressing their need for acknowledgment and support.

Many immigrant informal caregivers expressed the need for access to information available in their native language, as they encountered difficulties when information was primarily provided in languages they were less proficient in, such as Swedish [50]. In addition to language barriers, immigrant informal caregivers also expressed concerns about the trustworthiness and reliability of the information they found [50,51]. Collaborative efforts among health care organizations, community support services, and culturally diverse caregiver networks can contribute to the development and dissemination of culturally sensitive information resources in multiple languages [59]. In our study, we use the “trustworthiness” design principle. Previous research has indicated that the trustworthiness of the information presented is an important concern for users [60]. Addressing this concern can increase the persuasiveness of the app.

One prominent issue identified in our study is the lack of informal support and the burden of sole responsibility carried by immigrant informal caregivers, particularly female caregivers. Many immigrant informal caregivers expressed the need for additional support from their family members or friends, especially when they have other obligations, such as studying at the university or working. This finding aligns with studies emphasizing the multiple roles and responsibilities that immigrant women often fulfill within their families [49,61]. These caregivers feel overwhelmed by the demands of caregiving, which can hinder their ability to engage in other important activities or maintain a healthy balance in life. To address this issue, most immigrant informal caregivers suggested implementing joint coaching sessions involving family members, health care providers, and the primary caregiver. These sessions aimed to create a supportive network that can share the caregiving responsibilities and provide respite for the primary caregiver. By actively involving family members in discussions about caregiving responsibilities, challenges, and needs, they can gain insights into the daily struggles faced by the primary caregiver. This increased awareness can promote shared responsibility and encourage family members to provide support and assistance when needed [49,61]. Health care providers play a crucial role in facilitating these joint coaching sessions. They can provide guidance, practical advice, and access to appropriate resources. It is important to note that these joint coaching sessions should be culturally sensitive and linguistically appropriate to ensure effective communication and participation among all stakeholders [49,50,52].

Our findings indicate that most immigrant informal caregivers experienced difficulty accessing doctors or having medical assistance on time. The difficulties immigrant informal caregivers face in accessing timely health care services emerge as a recurring theme across several studies [48,50,52]. The literature emphasizes the importance of providing timely and efficient health care services to caregivers and their care recipients [49,51]. By addressing the challenges immigrant informal caregivers face in accessing health care, health care systems can better support these caregivers in their caregiving role. Accessible and culturally sensitive services can contribute to their ability to manage their care recipient’s health needs effectively and enhance their overall well-being. Another significant barrier to accessing appropriate health care services faced by immigrant informal caregivers is their limited understanding of their host countries’ health care and social welfare systems in their host countries [48,61]. Navigating complex health care systems becomes particularly challenging for these caregivers, who may come from different cultural backgrounds with different health care practices and beliefs. This lack of understanding is further exacerbated by comparisons made with the health care systems in their home countries, which are often perceived as more familiar and relevant to their needs [62]. Health care professionals and policy makers should recognize and address the unique needs and challenges faced by immigrant informal caregivers. Culturally sensitive approaches, targeted information and support, interpreter services, and simplified explanations of the health care system and insurance policies can help alleviate the difficulties faced by immigrant informal caregivers in accessing and navigating health care services [59]. Existing research on persuasive design suggests that design principles such as “social comparison” and “competition” are commonly favored in similar apps [60]. However, considering that caregivers are vulnerable users, implementing these design principles may exacerbate their stress levels. In this regard, this study proposes an alternative approach, advocating for “social learning” as a means for caregivers to collaboratively share and learn from each other’s caregiving experiences in their caregiving journey.

### Limitations and Future Research

This study’s findings should be considered in light of these limitations. First, we proposed an extended PSDM called PSD-I. A limitation of this study is that this extension is based on interviews with a group of immigrant caregivers that may not be fully representative, as we mostly received participants from the Middle Eastern region. More empirical evidence to explore the implications of PSD-I could contribute to the validity of this model in other contexts. In our study, we observed no discernible differences in caregiver needs when considering the age of the care recipient or the nature of the condition being cared for. Hence, we have put forth design recommendations for a general e-coaching app, albeit acknowledging that this approach may restrict the depth of information and coaching available compared with an e-coaching app tailored to a particular condition. As a result, future research efforts should concentrate on developing specialized e-coaching apps catering to specific caregiving scenarios, such as those involving young adult caregivers or care recipients. In addition, there are other caregiving contexts, such as stroke and cancer care, which we were unable to include in our study because of the unavailability of relevant caregiver data. Subsequent research endeavors could aim to improve the inclusivity of these underrepresented caregiving scenarios. Self-selection bias may also affect the sample’s representativeness, as those who are more motivated to participate may have different experiences or needs than those who do not participate. To gain a more comprehensive understanding of the needs and experiences of immigrant informal caregivers in Sweden, further research with a larger sample size is needed. Further research is needed to determine the applicability of these results in other populations and settings.

### Conclusions

In this study, we shed light on the significant needs of immigrant informal caregivers and provide insights for the design of mHealth apps in the caregiving domain. Our findings lead to several key conclusions regarding the needs of immigrant informal caregivers. First, it is apparent that these caregivers would greatly benefit from training programs designed to prepare them to care for their patients. This would help them develop the necessary skills and knowledge to perform their caregiving duties effectively. Second, the study found that many of these caregivers struggled with understanding the Swedish language and culture and therefore needed assistance. Third, the use of mHealth apps to access information and services is an area where these caregivers need support, as they may not be familiar with these systems. Fourth, the study revealed that many of these caregivers lacked recognition and appreciation for their work, which is a concern that needs to be addressed. Finally, access to relevant information about caring for their patients is essential for these caregivers, as it can help them provide better care and support. In addition, support is needed to help caregivers manage the stress and mental health impact of caring, as it can significantly impact their well-being and ability to perform their duties.

This study also proposes an extended and adapted PSDM for informal caregiving called PSD-I by including persuasive design principles of “facilitating conditions” and “verbal encouragement.” This adapted model provides a structured approach for designing interventions that effectively support and empower informal caregivers in their caregiving roles. By applying the design principles of PSD-I, we present the design of a mobile e-coaching app that addresses immigrant informal caregivers’ specific needs and challenges in Sweden. We also describe the implementation of these PSDM design principles for developing the e-coaching prototype. This prototype is a practical demonstration of how the PSD-I model can be effectively used to create tailored interventions for informal caregivers. PSD-I may serve as a resource for researchers and designers aiming to create impactful digital apps and services specifically tailored to the needs of the caregiving population.

## Appendix

Multimedia Appendix 1 COREQ (Consolidated Criteria for Reporting Qualitative Research) checklist.

Multimedia Appendix 2 Interview guide.

## Abbreviations

ADHD: attention-deficit/hyperactivity disorder
COREQ: Consolidated Criteria for Reporting Qualitative Research
FAQ: frequently asked question
PSDM: Persuasive System Design Model
